# Neonatal Simulation Program: A 5 Years Educational Journey From Qatar

**DOI:** 10.3389/fped.2022.843147

**Published:** 2022-03-21

**Authors:** Mohammad A. A. Bayoumi, Einas E. Elmalik, Hossamaldein Ali, Sunitha D'Souza, Jojo Furigay, Ava Romo, Sunitha Shyam, Rajvir Singh, Olfa Koobar, Jihad Al Shouli, Matheus van Rens, Fouad F. Abounahia, Ashraf Gad, Mostafa Elbaba, Samawal Lutfi

**Affiliations:** ^1^Neonatal Intensive Care Unit (NICU), Women's Wellness and Research Center (WWRC), Hamad Medical Corporation (HMC), Doha, Qatar; ^2^Pediatric Department, Hamad General Hospital (HGH), Hamad Medical Corporation (HMC), Doha, Qatar; ^3^Medical Research Center, Hamad Medical Corporation (HMC), Doha, Qatar

**Keywords:** newborn infant, curriculum development, neonatal simulation program, simulation-based education, healthcare simulation, mastery learning

## Abstract

We describe the process of implementation, adaptation, expansion and some related clinical intuitional impacts of the neonatal simulation program since its launch in 2016 in a non-simulation neonatal unit. The team has developed 6 types of curricula: 1 full-day course and 5 half-day workshops. A total of 35 free of charge simulation courses/workshops were conducted, 32 in Qatar and 3 abroad with a total of 799 diverse participants. There was a steady increase in the overall success rate of PICC insertion from 81.7% (309/378) to 97.6% (439/450) across 3 years (*P* < 0.0001). The first attempt PICC insertion success rate has been also increased from 57.7% (218/378) to 66.9% (301/450) across 3 years. The mean duration of PICC insertion has been improved from 39.7 ± 25 to 34.9 ± 12.4 min after implementing the program (*P* = 0.33). The mean duration of the LISA catheter insertion at the beginning of the workshop was 23.5 ± 15.9 compared to 12.1 ± 8.5 s at the end of the workshop (*P* = 0.001). When it came to clinical practise in real patients by the same participants, the overall LISA catheter insertion success rate was 100% and the first attempt success rate was 80.4%. The mean duration of LISA catheter insertion in real patients was 26.9 ± 13.9 s compared to the end of the workshop (*P* = 0.001). The mean duration of the endotracheal intubation at the beginning of the workshop was 12.5 ± 9.2 compared to 4.2 ± 3.8 s at the end of the workshop (*P* = 0.001). In real patients, the first-attempt intubation success rate has been improved from 37/139 (26.6%) in the first year to 141/187 (75.5%) in the second year after the program implementation (*P* = 0.001). The mean duration of successful endotracheal intubation attempts has been improved from 39.1 ± 52.4 to 20.1 ± 9.9 s (*P* = 0.78). As per the participants, the skills learned in the program sessions help in protecting neonates from potential harm and improve the overall neonatal outcome. Implementing a neonatal simulation program is a promising and feasible idea. Our experience can be generalised and replicated in other neonatal care institutions.

## Introduction

Numerous factors contribute to neonatal morbidity and mortality, and insufficient experience of neonatal healthcare providers managing crises is one major cause worldwide (1). Neonatal emergency, procedures and resuscitation scenarios are usually complex, challenging, requiring excellent cognitive skills, communication and teamwork between and within the multidisciplinary neonatal healthcare teams handling such cases (2, 3). Practical procedures, neonatal resuscitation skills, critical thinking, communication skills, and effective team dynamics are integral to neonatal care. These domains are essential for the core paediatric speciality and neonatal subspecialty progress curriculum for trainees and established experienced neonatologists (4).

Old-fashioned “trainee” models in medical education do not offer as many opportunities to practise clinical procedures and face hot complex neonatal real-life situations on real patients. Patient safety is a top priority all over the world hence it has resulted in a shift to simulation-based learning (5). Most of the trainees and many mid-career paediatricians join the neonatal speciality with minimal prior exposure to life-saving cognitive and psychomotor skills. Many paediatric residents, unfortunately, finish their education without completing the necessary practical competence and resuscitation expertise in the care of neonates mostly due to non-participation (6, 7). With recent changes in junior and mid-career physician's contracts, reduced working hours and evolving clinical practises, such as favouring less invasive forms of ventilation and surfactant administration worldwide, trainees may not get enough clinical exposure to gain these rare but pivotal skills. As DeMeo et al. further explore, neonatal endotracheal intubation for example remains a critical skill for paediatric residents and neonatal-perinatal fellows in the NICU, but they don't usually get a lot of chances to learn this vital skill (6). Simulation hence remains one of the ways to enhance this vital competency (8, 9).

Simulation-based education is an excellent way of practising problem solving, decision making, understanding human factors, critical thinking skills under pressure, communication reflecting great teamwork and even identifying ergonomics challenges, all of which will ensure better patient care and reduce the risks associated with the daily critical neonatal practise (10). It is considered a safe bridge between theory and practise for this infrequently occurring clinical event. Creating a robust foundation in simulation training, implementing a collaborative, multidisciplinary approach of the healthcare staff, and initiating a quality improvement agenda were important goals leading to the establishment of a Neonatal Simulation Program launched in September 2016 at the Hamad Medical Corporation (HMC).

## Program Objectives

The main goal of the Neonatal Simulation Program is to improve patient safety by creating a safe and realistic environment in which all clinical neonatal professionals, be it novice or seasoned, can hone their skills, practise new and advanced techniques, and develop their clinical competencies. This is important, especially for competencies like neonatal intubation, which might be more rarely required in the field, but is a skill of vital importance with no room for errors in the real world. Results from other studies indicate that training models designed for adult practises cannot be readily transferred to neonatal skills without major changes made to the training models (11–15).

By launching the program, we aim to demonstrate, implement and disseminate unique high-quality neonatal simulation-based experiences to participants all over the world. We wanted to encourage enhanced involvement of clinicians, thus making full use of this available training to upgrade their mastery learning and clinical performance. Transferring skills learned in simulation sessions to the bedside might be a challenge. One of the goals of the program is to fill in the gap between the simulation environment and real-life scenarios (16). Another goal of this program was to provide simulation training free of cost, at least at the initial phase, to encourage more participation and increase awareness.

Our objectives align with the HMC's Strategic plan in the establishment of collaboration with partners across medicine. That will be positively reflected in clinical patient care and safety which is our goal.

## Methods

The Women's Wellness and Research Center (WWRC) is the main hospital for women and newborns health services in Qatar. The hospital lies under the umbrella of Hamad Medical Corporation (HMC), Doha, Qatar. WWRC is a tertiary teaching hospital that accommodates more than 18,000 deliveries per year and has a level III NICU with 112 beds. The data were collected retrospectively from WWRC only after getting the Institutional Review Board (IRB) approval from the Medical Research Centre (MRC) in Hamad Medical Corporation (Protocol Number: MRC-01-22-060).

Neonatal practise is an example of crisis resource management where multi-disciplinary teams work together to provide the best possible care for highly vulnerable neonates. It was noted that there is a considerable performance gap in the neonatal team responses, especially in critical situations. As simulation-based education is the best way in learning and enhance team dynamics in such situations, the neonatal simulation team has been launched in September 2016 to enhance the NICU team's performance in critical unusual situations. The neonatal simulation team first designed the neonatal emergencies simulation course in 2016, then the in-situ neonatal simulation workshop in 2017, the neonatal golden hour simulation workshop in 2019, the less invasive surfactant administration simulation workshop in 2020, and the neonatal transportation simulation workshop in 2021.

The neonatal simulation steering committee was formulated from different subspecialties to lead this program. The core team includes two neonatologists, one paediatric nephrologist, two clinical pharmacists, one respiratory therapist, one NICU nurse as well as one nurse educator. Three of the members are internationally accredited in healthcare simulation and medical education. Of those, two hold positions in the Society of Simulation in Healthcare (SSH) and the International Pediatric Simulation Society (IPSS).

**Figure 1:**
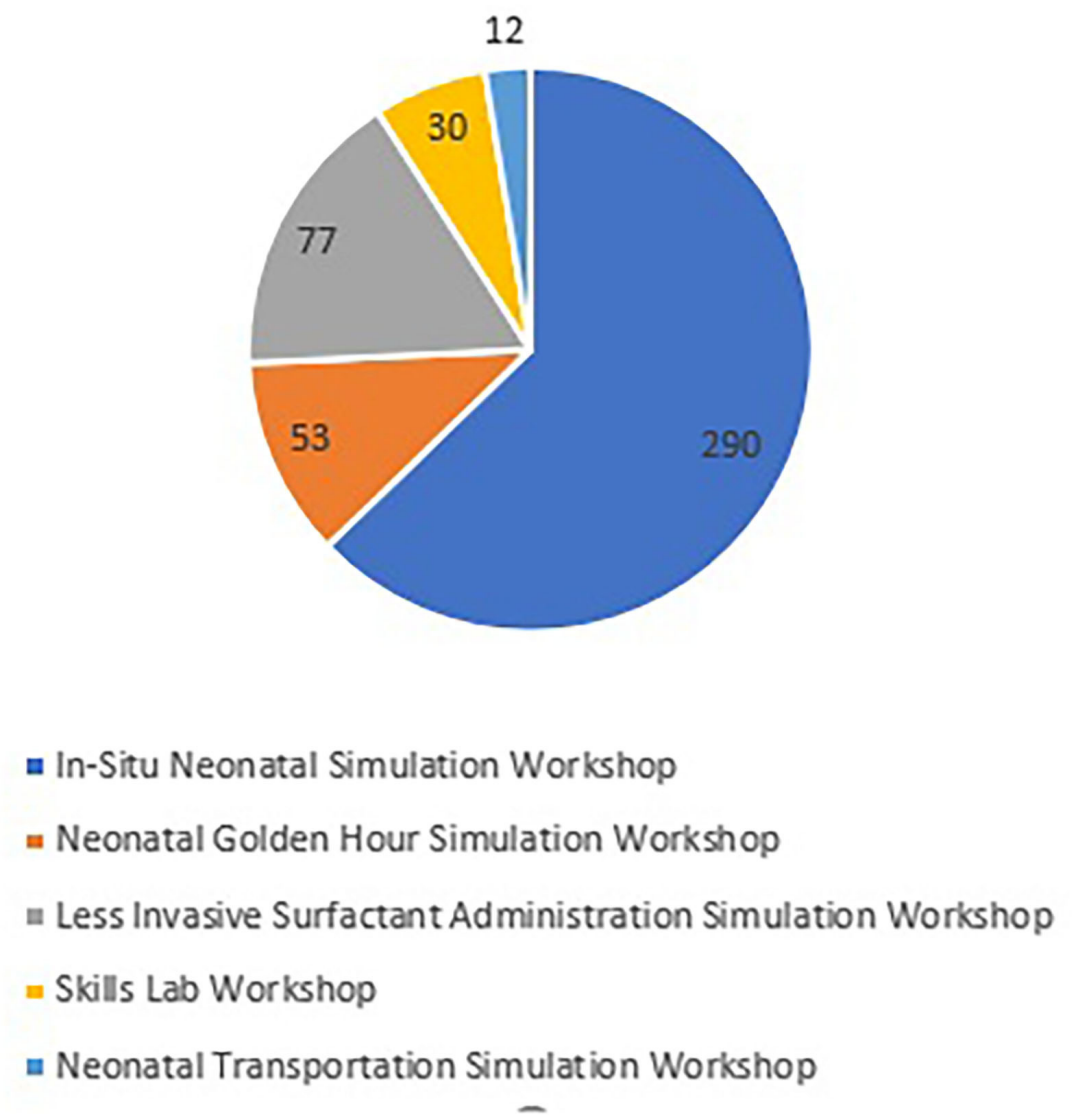
Indicates the number of attendees for the 5 half-day workshops.

**Figure 2:**
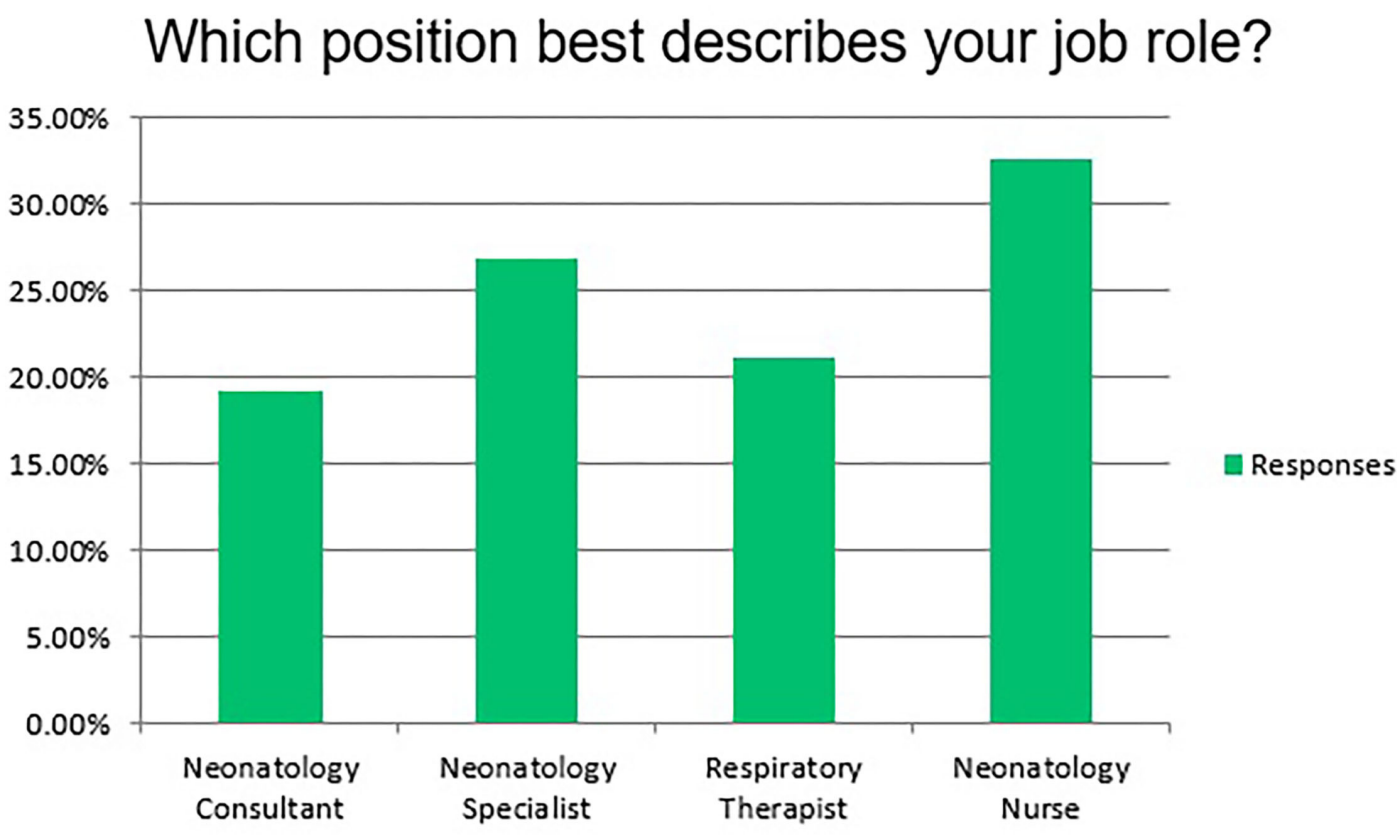
Indicates the categories of the participants.

After setting the scope, performing the educational needs assessment, and designing the learning objectives, the team has developed 6 types of curricula; 1 full-day course and 5 half-day workshops. Thirty Five free of charge simulation courses/workshops were conducted in Qatar and abroad based upon the educational need's assessment. The full-day course is the neonatal emergencies simulation course which was conducted 8 times; 5 in Qatar and 3 abroad in Egypt, UAE, and Singapore. In addition to that, 27 half-day workshops were conducted in Qatar: 13 *in situ* neonatal simulation workshops, 7 less invasive surfactant administration simulation workshops, 2 neonatal golden hour simulation workshops, 1 neonatal transportation simulation workshop as well as 4 skills lab workshops. The program non-mandatory sessions attracted 799 local, regional and international attendees. We have a total of 46 current facilitators for all the courses/workshops in addition to 4 who already left the program. The team has produced 7 educational videos for different courses and workshops to aid in the educational process. The full-day neonatal emergencies simulation course attracted a total of 337 local, regional and international participants. Figure 1 shows the number of attendees of the 5 half-day workshops. Figure 2 indicates the percentages of professions of the participants.

All courses and workshops were approved by the HMC Medical Education Department and were accredited by the Department of Healthcare Professions (DHP) in the Ministry of Public Health (MOPH) with both category I and category III Continuous Professional Development (CPD) hours. The team prepared the required equipment and conducted the first simulation workshops in the Hamad Medical Corporation's Itqan Clinical Simulation and Innovation Center as well as in Sidra Medicine Simulation Center. The logistics of equipment and catering services for the workshops were all supplied by the supporting departments of the Corporation; venues were booked well ahead (6 months to a year) of the planned schedules.

A post-event online survey questionnaire (SurveyMonkey®) was specifically designed for each program curriculum. It was sent to all the participants a few days after the events and it was made mandatory to fill to get the CPD certificate. The survey questions were preliminarily piloted by some learners and facilitators to test the validity, reliability and time needed to fill. The surveys included personal demographic data, venue, facilitator preparation and attitudes, cognitive psychomotor and behavioural skills acquired in the events as well as suggestions and recommendations for improvement.

### Neonatal Emergencies Simulation Course

The neonatal emergencies simulation course is a full day course that has 6 complex multi-phasic advanced hands-on neonatal scenarios. In this course, we used high-fidelity manikins (SUPER TORY® S2220, Gaumard). Due to its clinical importance, neonatal endotracheal intubation was practised by the candidates in 3 of the 6 stations. Umbilical venous and arterial catheterization, peripherally inserted central catheterization, chest tube insertion, and lumbar puncture were also practised in different course stations by the participants to promote and maintain their cognitive, psychomotor and behavioural skills at the optimum level. Post-simulation scenario team debriefing was conducted in each station.

### Less Invasive Surfactant Administration Simulation Workshop

Due to the current evidence, Less Invasive Surfactant Administration (LISA) has been recently introduced to our day to day clinical practise. Before the implementation, the neonatal simulation team has conducted 7 half-day less invasive surfactant administration simulation workshops. In this workshop, we used the surfactant administration catheter (Surfcath - Vygon Ltd, UK). The accredited workshop has a didactic session, procedural hands-on practise using a medium-fidelity manikin, as well as a debriefing session. The team has ensured that the participant has been skilled enough to practise the procedure in a real clinical situation (17).

### Neonatal Golden Hour Simulation Workshop

Extremely low birth weight infants are prone to hypoglycemia, hypotension, and hypothermia after delivery. The neonatal simulation team has developed the neonatal golden hour simulation workshop aiming to reduce the rates of these neonatal morbidities and optimise and standardise the evidence-based clinical care practises during the first hour of life. In this workshop, we used the incubator (Giraffe Incubator Carestation, GE Healthcare, United States) ventilator (Dräger Babylog® VN500), manikin (Premature Anne, Laerdal Medical), and all the other equipment used in the neonatal resuscitation program course. This workshop has a didactic session with video materials, a complex timed preterm inter-professional resuscitation scenario using a medium-fidelity manikin, followed by a post-resuscitation team debriefing (18, 19).

### Skills Lab Workshop

Rare neonatal procedures might be very challenging even for experienced neonatologists. This might be due to its difficulty and being very rare to face in real life. To obtain and maintain the staff privileges for these procedures, the NICU deputy director has designed this half-day workshop to practise the exchange transfusion, peripheral arterial line insertion, abdominal paracentesis, intraosseous needle insertion, and pericardiocentesis (20, 21).

### Neonatal Transportation Simulation Workshop

The neonatal transportation program in HMC is considered the first retrieval program in the region with more than 1,100 local, regional and international transports. Safe local, regional and international neonatal transportation requires a highly reliable and efficient inter-professional transportation team that is expert enough to provide advanced neonatal care in unusual and resource-limited situations. Providing didactic and experiential learning alone has been proved to be insufficient to fully prepare the interprofessional neonatal transportation teams that have limited exposure to rare unexpected events. Simulation-based education has been proved to enhance and maintain knowledge, skills, and the experiences of different interprofessional transportation team members (5, 22). The workshop has a video-assisted didactic part, 4 hands-on sessions using medium and high fidelity manikins and finally a debriefing session (5).

### *In-situ* Neonatal Simulation Workshop

*In-situ* neonatal simulation workshop has been conducted by the team in the NICU many times. The content and the scenario varied each time based upon the educational needs assessment and staff requests. This included communication skills scenarios, conflict resolutions, resuscitation skills monitoring, debriefing skills, procedural skills, team responses and inter-professional team development (4, 23, 24).

### Statistical Tool

Descriptive statistics in the form of mean and standard deviations for interval variables and frequency with percentages for categorical variables were calculated. Chi-square tests were applied to see the association between the two categorical variables. Unpaired Student *t*-tests or Mann Whitney U tests as appropriate were applied to compare interval variables between the two categories. Paired student t-tests were applied to see a significant difference in mean durations of LISA catheter insertion at the beginning versus the end of the LISA simulation workshop and between the end of the workshop and the duration in real patients. Additionally, the paired student t-tests have been used to see a significant difference in the mean duration of endotracheal intubation at the beginning versus the end of the neonatal intubation simulation workshop. *P*-value 0.05(two-tailed) was considered a statistically significant level. SPSS 28.0 statistical package was applied for statistical analysis.

## Results

The courses and workshop gradually increased in number and quality based upon the participant's requests as well as the needs assessment.

Simulation-based education has since become a routine part of the unit's educational activity over the last years (Figure 1). Overall, the program has been well received by the participants. The data obtained from the post-event survey each session was analysed. Overwhelmingly positive feedback was received with suggestions for improvement and some requests. Candidates derived generic learning points and participants' self-reported data from surveys include improved targeted communication skills, clear leadership roles, role clarity, confidence, teamwork, knowledge sharing within the team, raising concerns, constructive intervention, and regular reflection and re-evaluation. In the post-event surveys, the participants commented that the program has helped enrich their technical, emotional and critical thinking skills, which they believe will help them to transfer those skills in a real clinical environment. These frequent courses/workshops gave the candidates the full chance to practise more and to master their technical, cognitive, and psychomotor skills (25, 26). Each course/workshop has been repeated many times as per the educational needs assessment and participants' requests. Conducting the program sessions, facing unexpected situations, timely preparation, and using the participants' and facilitators' feedback has improved the session's quality over time and decreased the undesired events.

## Program Impacts on Clinical Practise

Simulation is considered as the safe bridge between theory and clinical practise. The aim of neonatal simulation-based education is not only to learn and maintain new skills but also to enhance and ensure the soft transferability of these skills from simulation to clinical practise. The success of any simulation program depends on its ability to bridge the gap between simulation and clinical practise (26, 27). We are reporting some clinical impacts of the program on neonatal clinical practise. More detailed clinical outcomes for each course/workshop are beyond the scope of this article.

**Figure 3:**
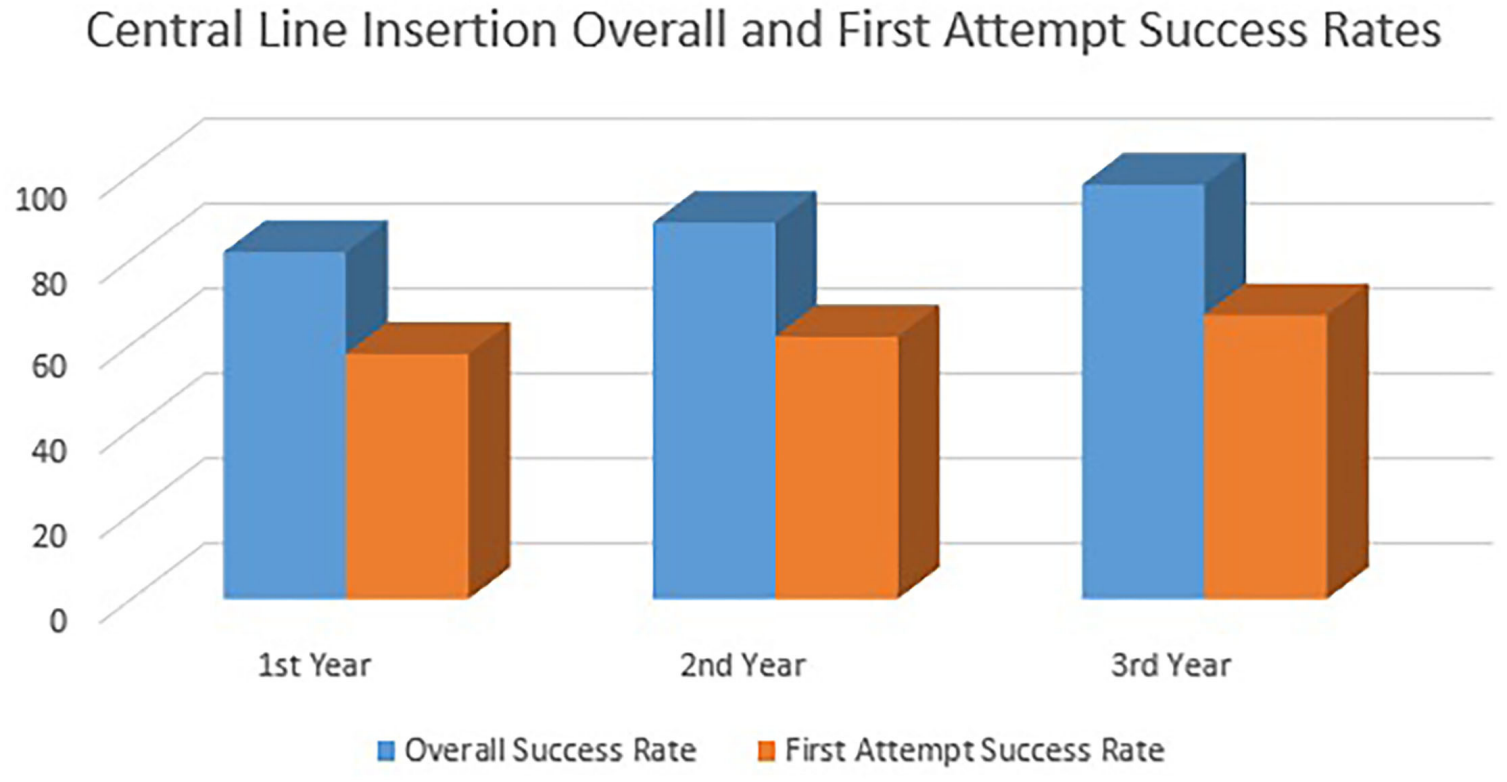
Indicates the central line insertion overall and first attempt success rates.

We traced the overall and first-attempt success rate of the Peripherally Inserted Central Catheters (PICCs) in the first 3 years after conducting the central line simulation workshops. Figure 3 shows the gradual sustained improvement in both overall and first-attempt success rates across the 3 years.

The mean duration of PICC insertion has also been improved from 39.7 ± 25 to 34.9 ± 12.4 min after implementing the central line simulation workshops (*P* = 0.33). This duration was measured from the start of the procedure scrubbing till removing the personal protective equipment. A duration of 24.1 min is reported in the literature (28).

Another highlighting impact of our program is the Less Invasive Surfactant Administration (LISA) which has been introduced to our neonatal practise in 2020. The team has conducted 7 simulation workshops for the healthcare providers involved in this procedure. LISA Catheter insertion is a critical skill that might be challenging for learners to acquire and master within the time frame of 30 s even for those who attended the simulation workshop. One of the workshop aims was to let the participants practise the procedure as much as possible to get their confidence to do it in real patients using the rapid cycle deliberate practise (29, 30). The mean duration of the LISA catheter insertion by the participants at the beginning of the workshop was 23.5 ± 15.9 s and the end was 12.1±8.5 s after completing the rapid cycle deliberate practise (*P* = 0.001). When it came to clinical practise in real patients by the same participants, the overall LISA catheter insertion success rate was 100% and the first attempt success rate was 80.4%. The mean duration of LISA catheter insertion in real patients was 26.9 ± 13.9 s compared to the end of the workshop (*P* = 0.001).

**Figure 4:**
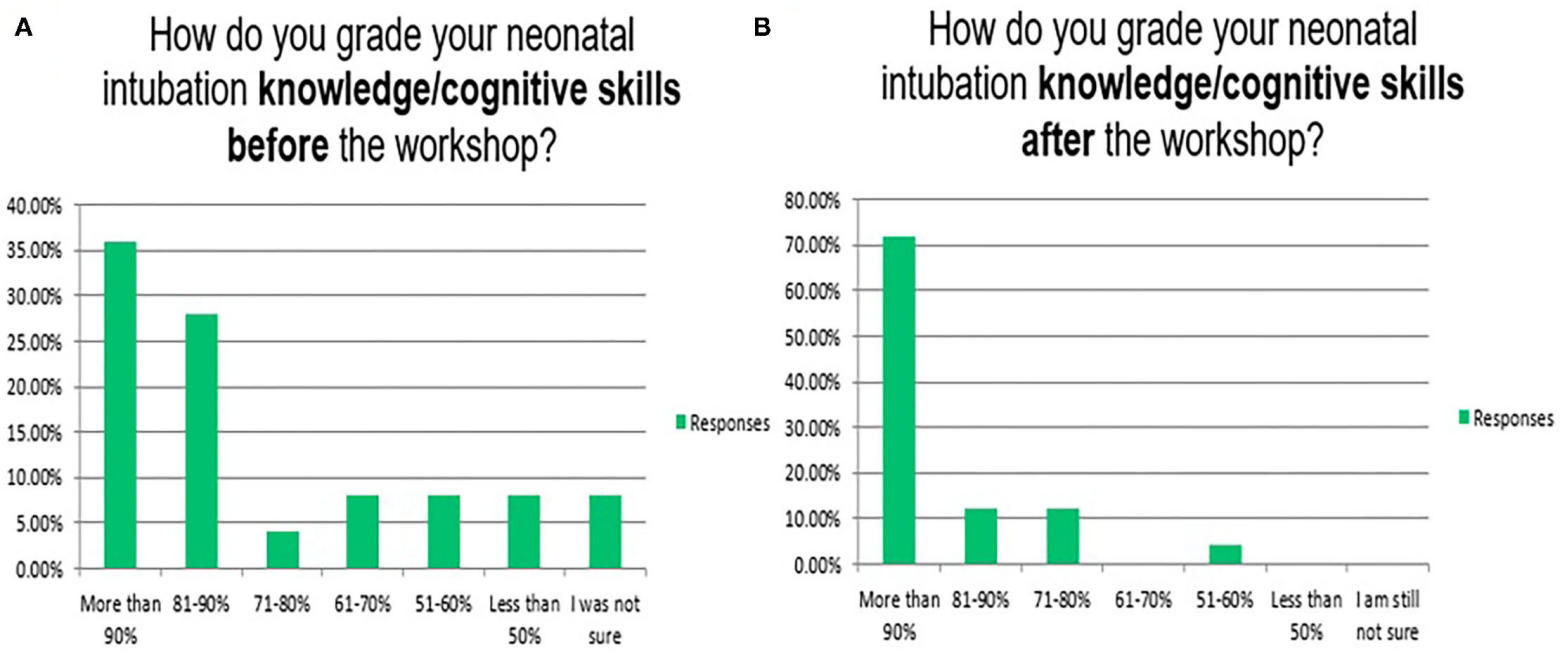
Reflects the attendees' perception/opinion of their knowledge/cognitive skills of the neonatal endotracheal intubation before **(A)** and after **(B)** the simulation event.

**Figure 5:**
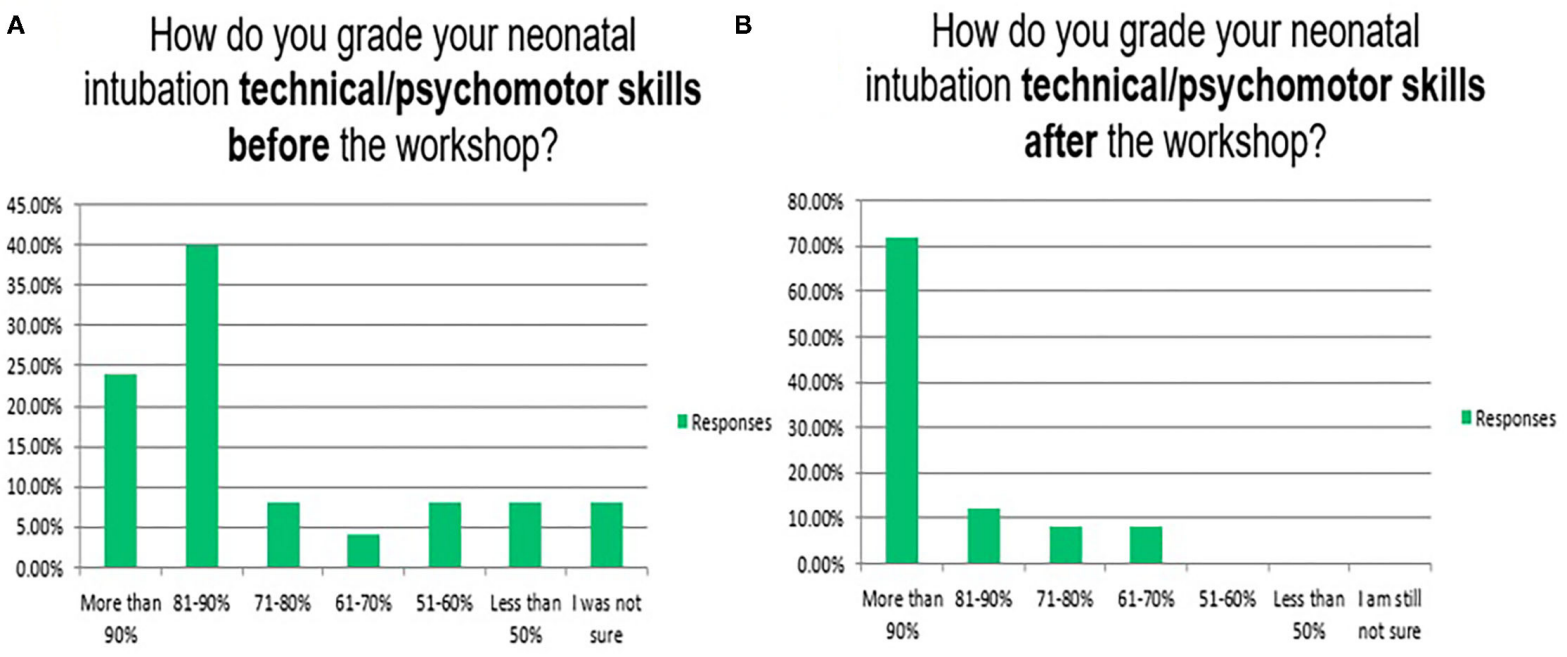
Reflects the attendees' perception/opinion of their technical/psychomotor skills of the neonatal endotracheal intubation before **(A)** and after **(B)** the simulation event.

**Figure 6:**
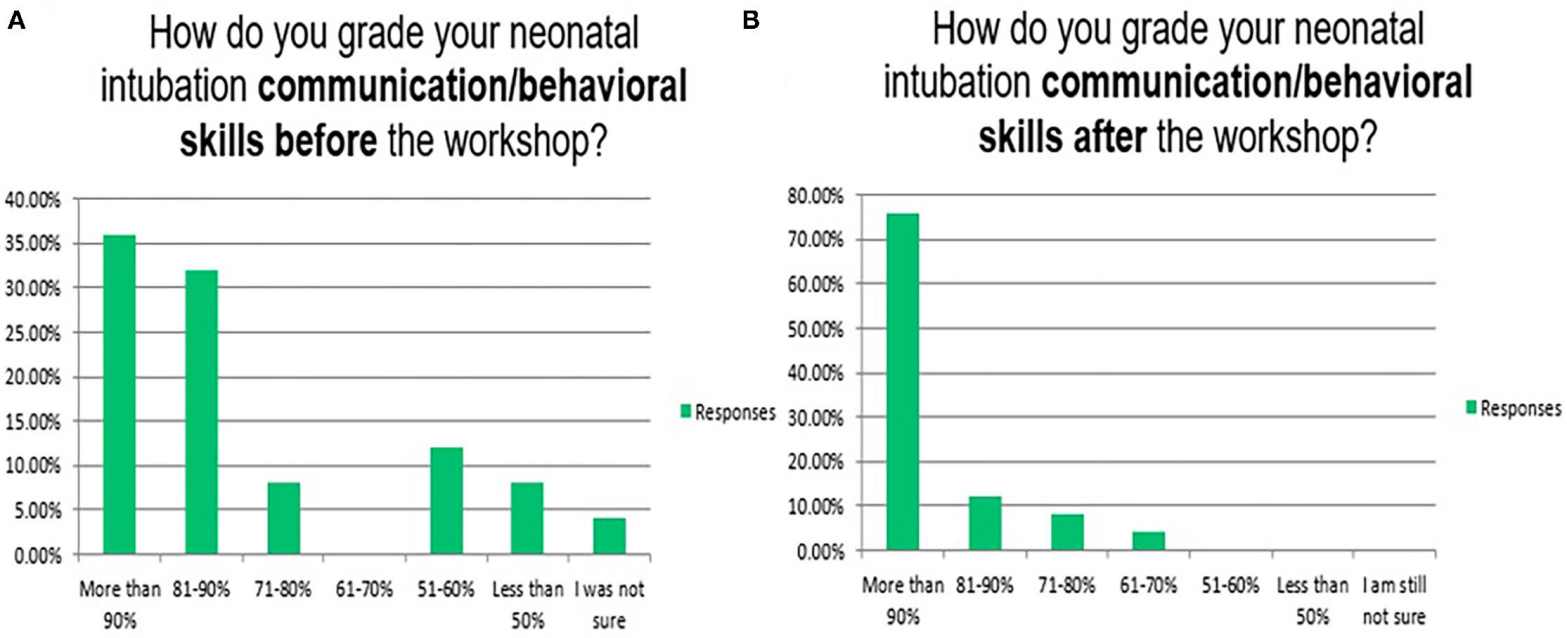
Reflects the attendees' perception/opinion of their communication/behavioural skills of the neonatal endotracheal intubation before **(A)** and after **(B)** the simulation event.

**Figure 7:**
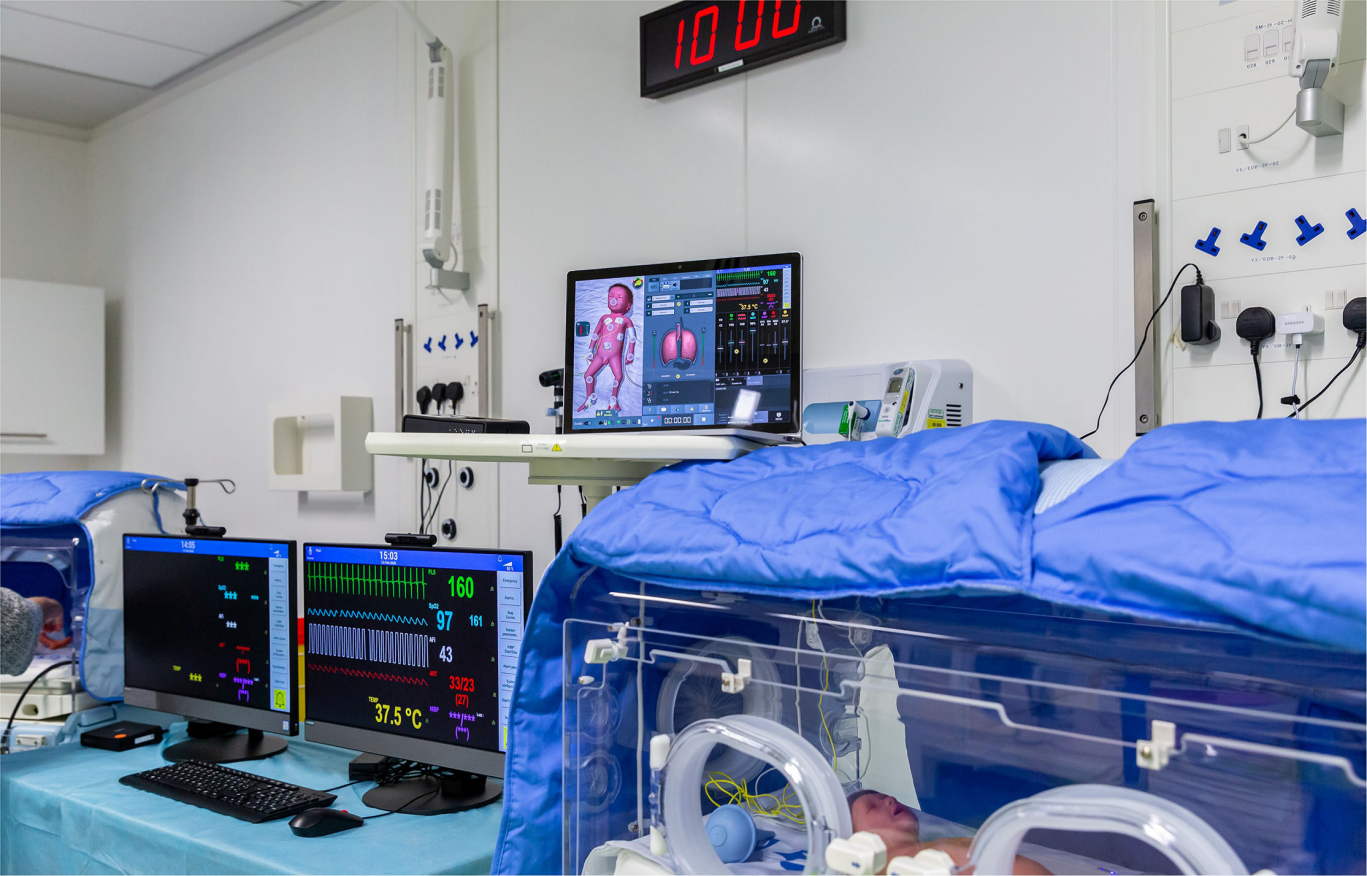
High-fidelity manikin and incubator used in neonatal simulation program events.

**Figure 8:**
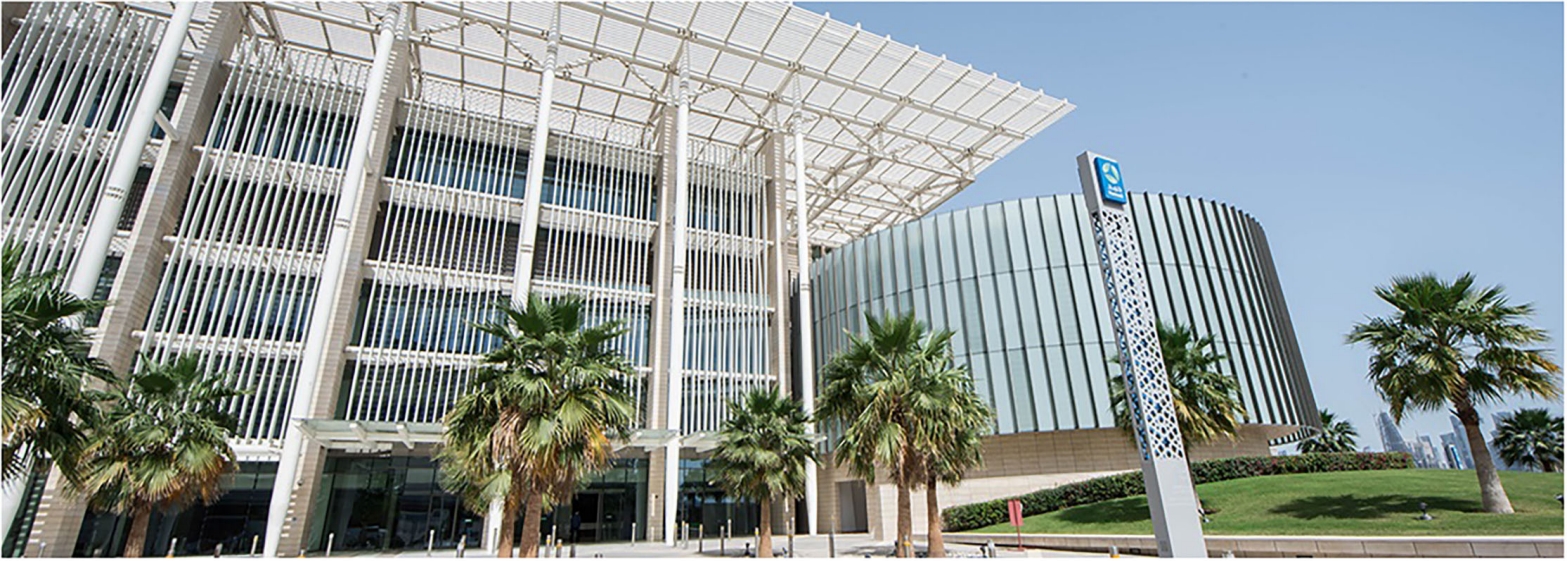
Itqan clinical simulation and innovation centre in hamad medical corporation.

Neonatal endotracheal intubation is a crucial procedure (31). In our program, the knowledge/cognitive, technical/psychomotor, and communication/behavioural skills for neonatal endotracheal intubation have been taught in 3 of the 6 complex interprofessional simulation scenarios/stations of the neonatal emergencies simulation course as well as in the *in-situ* neonatal simulation workshop (32). Figures 4–8 reflect the attendees' perception/opinion of their 3 learning domains of the neonatal endotracheal intubation before and after the simulation event.

The mean duration of the endotracheal intubation by the participants at the beginning of the workshop (12.5 ± 9.2 s) and the end (4.2 ± 3.8 s) after completing the rapid cycle deliberate practise (*P* = 0.001). In real patients, the first-attempt intubation success rate has also been improved from 37/139 (26.6%) in the first year to 141/187 (75.5%) in the second year after the program implementation (*P* = 0.001). The mean duration of successful endotracheal intubation attempts has been improved from 39.1 ± 52.4 to 20.1 ± 9.9 s (*P* = 0.78). Other institutions reported 49, 50, 60, and 64% as first attempts success rates. It is highly operator dependent (8, 22, 33). It is important to mention that most of our tracheal intubations are performed by neonatal-perinatal fellows and advanced neonatal providers e.g., specialists and consultants rather than the trainee paediatric residents. This explains the gradual decrease in opportunities for trainee paediatric residents to perform tracheal intubation in their neonatology rotations (19).

The Neonatal Resuscitation Program (NRP) recommendation for laryngoscopy and intubation duration is <30 s starting from the introduction of the laryngoscope (30). However, there is a difference in the procedural time between the simulation at the end of the workshop and the clinical practise in both LISA catheter and ETT insertions. This procedural time difference might be explained by the slipperiness, secretions, and softer textures of babies compared to the mannequins. Moreover, the interprofessional nature and stressors of the NICU clinical environment play a role in creating this procedural time difference (33). One of our program goals is to narrow the performance gap between simulation and practise.

## Discussion

This program is the first of its nature in Qatar. The growing population in the country and region creates a need for the establishment of more neonatal simulation programs.

This program was created to improve quality, patient safety and provide the safest, most effective and compassionate care. These were effectively demonstrated by:

- Development of clinical situational approaches based on quality and patient safety improvement.
- Enhancement of multi-disciplinary teamwork.
- Demonstration and proof of improvement.

The key features of the success of program implementation were staff dedication, hard work, persistence, sacrifices, and discipline. The team has volunteered their time, money and effort to get successful. We faced many rejections, criticism, doubts, failures, complaints and we were able to overcome them. This can be attributed to careful planning with systematic execution, positive thinking, continuous professional development, collaborating available experience, administrative support, and extensive regular training of the staff before, during and after activation. Inter-Professional Education allowed the instructors and the participants to learn with, from, and about each other.

Our results show a sustained improvement in different neonatal procedural skills including PICC insertion, LISA, and endotracheal intubation overall and first-attempt success rates. By the end of our program sessions, the times needed to perform the procedures have been significantly shortened. However, there is still a gap between the simulation and clinical scenarios in real patients and retention of the acquired skills in simulation sessions remains a challenge. Despite the ongoing effort of enhancing the realism, psychological, physical, conceptual and environmental fidelity, the appearance, texture, and function of the manikins are still different enough from the real patients. This might be contributed to the confounders and stressors usually present in the clinical environment leading to a performance gap between the simulation and real patients (33, 34).

One of the biggest challenges was attracting interested attendees due to the lack of simulation culture in Qatar at that time. Not all staff believed in simulation as a great tool for adult learning and practical experience and some said it is just an act! With continuous efforts to raise awareness about the importance of simulation-based education, our average attendance rose to 30 per workshop. The lowest number of attendees per event was 2 and the highest was 95.

Logistics was another challenge due to the varying numbers of attendees as well as the many training sessions planned. Although all arrangements and booking of required resources including auditoriums, were done months in advance, the organisers had to change plans to best fit the circumstances of the training day. For example, larger workshops of 95 attendees could not be accommodated in smaller training rooms as originally planned. Instead, the training was shifted to a large auditorium. Unfortunately, this also meant that not all participants could have a chance to practise certain procedures. To overcome this, a camera connected to the live screen was installed before the session and all the participants observed the scenario and procedure steps in detail on the screen. Different combinations of available rooms were tried during the different training sessions to provide the best learning environment for the attendees.

The lack of trained, fully dedicated facilitators was yet another challenge. Preparation for a full day simulation workshop is time-consuming, effort-intensive, expensive and stressful especially if there is no financial benefit for such volunteers and their simulation work is not recognised as part of their core work responsibilities.

Preserving session times and calendar bookings for facilitators and participants from different neonatal specialities was also a challenge in the busy NICU environment. Many of the supporting staff could not continue supporting the program due to clinical commitments, time constraints, and lack of financial benefits! To overcome this challenge, we tried to choose those who were fully dedicated to the pursuit of education and patient safety. However, this remains a big challenge!

The availability of the task trainers for some complex neonatal procedures was another challenge especially abroad. Examples include peripheral vascular access mannequin, lumbar puncture, umbilical catheterization and the chest tube task trainers. The mannequins and task trainers are expensive. As the need is the mother of invention, we created our task trainers for certain neonatal procedures. The task trainers include chest tube trainers, lumbar puncture task trainers, peripheral vascular access as well as the umbilical vascular access task trainer, made with lower technology but are with high fidelity.

Funding is the biggest challenge, and this is especially true when sessions are conducted outside of Qatar. The corporate has thankfully provided the venue, equipment, and caterings for the courses/workshops in Qatar. The additional cost for the courses/workshops conducted in Qatar only is QAR 31900 during the 5 years and that was all shouldered by the core team. It included courses filming and editing, camera and its accessories, stands, posters, special mannequin's maintenance and spare parts, online survey subscription, bells and stopwatches, and gifts for the best participants. For the 8 events conducted abroad, the cost included the flights' bookings, hotel's bookings, visas fee, and conferences registrations whenever applicable for 2-3 facilitators in each of the 8 events and that was also shouldered by the core team. We did not receive any support from the corporate or any of the simulation or pharmaceutical companies in the 5 years. Program sponsorships will enable the team to achieve more in the field of simulation-based education.

## Limitations and Future Directions

The main limitation is the retrospective nature of the study and the self-reported data from the participant's surveys.

Future goals of this program include:

- Designing validated assessment tools to help acquisition and assessment of different cognitive, psychomotor and behavioural skills.
- Establishing the frequency of simulation-based training/re-training to enhance and maintain the knowledge and skills for participants of different clinical expertise.
- Narrowing the gap between the performance in simulation and actual clinical performance.
- Increased use of simulation of various congenital abnormalities and enhancing its realism by the use of moulage.
- More tailoring of different complex non-resuscitation scenarios to mimic medical issues in the NICU as realistically as possible.
- Increased use of high-fidelity simulation is known to have a positive effect on learning.
- Expanding simulation beyond technical skill acquisition, conducting simulation research in human and system performance, ergonomics, and incorporating simulation into high-stakes skill assessments including leadership, risk and resources management are ongoing and pending tasks to complete our mission (35, 36).

We received invitations to conduct and replicate our simulation courses/workshops in Sudan, Egypt, Pakistan, Uganda, Rwanda, and Cambodia. Lifting the COVID-19 restrictions and program support will enable the team to move freely and conduct more sessions around the world.

The program will remain as the team believes that education is a continuous journey, not a destination, with much more to contribute to learning. We aim to expand by developing new curricula and by increasing the number of sessions of the already existing courses/workshops.

## Conclusions

Implementing a neonatal simulation program is a promising and feasible idea. Our experience can be generalised and replicated in other neonatal care institutions. The program provides support, resources, knowledge and encouragement. From our perspective, it facilitates reaching a considerable level of achievement within the main three pillars of health services; health, education and research.

There are ample research opportunities in neonatal simulation including research on different aspects of patient risk reduction, medical education, human factors, ergonomics, behavioural skills, patient safety, personnel training, communication within & between teams, as well as the universal application of new treatment practises and/or procedures. We hope to incorporate all these factors into future neonatal simulation research activities.

## Data Availability Statement

The original contributions presented in the study are included in the article, further inquiries can be directed to the corresponding author.

## Ethics Statement

Written informed consent was obtained from the individuals for the publication of any potentially identifiable images or data included in this article.

## Author Contributions

MB and EE contributed equally as co-first authors. They conceptualized, designed and founded the program, got the approvals and accreditations, did the educational needs assessment, designed the learning objectives, developed the program curricula, designed the scenarios, surveys and data collection instruments, coordinated and supervised data collection, paid the cost for all the program courses/workshops, drafted the initial manuscript, and reviewed and revised the manuscript. ME and SL contributed equally as co-senior last authors. They approved and supported the program, designed the neonatal emergencies simulation course, and the skills lab workshop respectively, designed data collection instruments, and critically reviewed the manuscript for important intellectual content. HA designed the neonatal emergencies simulation course and data collection instruments and reviewed the manuscript. MR, SD'S, JF, and AR helped in program logistics and equipment, collected data, and reviewed and revised the manuscript. OK conceptualized and designed the neonatal golden hour simulation workshop, and reviewed and revised the manuscript. JA conceptualized and designed the less invasive surfactant administration simulation workshop, and reviewed the manuscript. AG conceptualized, reviewed and supported the less invasive surfactant administration in the NICU and designed the data collection sheet for an *in-situ* neonatal simulation workshop. FA conceptualized and designed the neonatal transportation simulation workshop, and reviewed the manuscript. RS performed the statistical analysis. All authors approved the final manuscript and agree to be accountable for all aspects of the work.

## Alphabetic List of the Neonatal Simulation Program Instructors

Abdallah Kamal Hasan Mahmoud, Abdellatiff Hamdy Abdelwahab, Airene Lou Francia, Amr Moussa Khalil, Bader Kordi, Faisal Manakkal, Frances Martos, Grace Van Leeuwen, Irian Jade Linon Cabanillas, Joy Ann Rivera, Katherine Mariano, Kochumole Thomas, Krisha Garcia, Ma. Ana Princess Tisbe Villa, Mohammad Ayman Elkhateeb, Mohammed Gaffari, Nazla Mahmoud, Nestor Macaraeg, Nuha Abdelghaffar Nimeri, Ranilo De Guzman, Resmi Nair, Roderick Perdon, Roseline Soosai, Rosemary Rao Kotteswara, Safaa Alsayigh, Shafeeque Kunhi Abdullah, and Venkatesh Manjunath Shetthalli.

## Conflict of Interest

MB, EE, HA, SD'S, JF, AR, SS, RS, OK, JA, MR, FA, AG, ME, and SL are employed by Hamad Medical Corporation.

## Publisher's Note

All claims expressed in this article are solely those of the authors and do not necessarily represent those of their affiliated organizations, or those of the publisher, the editors and the reviewers. Any product that may be evaluated in this article, or claim that may be made by its manufacturer, is not guaranteed or endorsed by the publisher.

## Abbreviations

WWRC: Women's Wellness and Research Center
IRB: Institutional Review Board
HMC: Hamad Medical Corporation
NICU: Neonatal Intensive Care Unit
LISA: Less Invasive Surfactant Administration
PICC: Peripherally Inserted Central Catheter
CLABSI: Central Line-Associated Blood Stream Infection
ETT: Endotracheal Tube
NRP: Neonatal Resuscitation Program
DHP: Department of Healthcare Professions
MOPH: Ministry of Public Health
CPD: Continuous Professional Development
MRC: Medical Research Center.
